# Metastatic well-differentiated neuroendocrine carcinoma of the pancreas: case report and review of literature

**DOI:** 10.4076/1757-1626-2-8973

**Published:** 2009-09-01

**Authors:** Mersadies R Martin, Umer F Malik, Deepak Mohan, Ahmed Mahmoud

**Affiliations:** 1 Saint George’s University School of Medicine7405 Greenback Lane #109 Citrus Heights, CA 95610USA; 2 Department of Internal Medicine San Joaquin General Hospital500 West Hospital Road French Camp, CA 95231USA; 3 Department of Pathology San Joaquin General Hospital500 West Hospital Road French Camp, CA 95231USA; 4 Department of Surgery San Joaquin General Hospital500 West Hospital Road French Camp, California, CA 95231USA

## Introduction

Neuroendocrine carcinoma accounts for less than 5% of cancers of unknown primary site. When primary pancreatic cancer is diagnosed, 95% of cases are classified as aggressive adenocarcinoma and the remaining 5% of cases are caused by indolent behaving neuroendocrine carcinoma. We present a rare case of well-differentiated neuroendocrine carcinoma of the pancreas extending to the transverse colon, small bowel, stomach, and lymph nodes with metastasis to the caudate lobe of the liver. The patient had a two-year history of midepigastric abdominal pain that eventually sent him to the Emergency Department and after extensive tests we removed the entire tumor with margins free of growth. Included in this case report are many illustrations to show the severity and we further emphasize the importance of examining patients thoroughly especially when vague symptoms are chronic.

## Case presentation

A 58-year-old African American male presented to the Emergency Department by ambulance complaining of a constant, sharp, and worsening midepigastric pain that radiated posterior for approximately 30 minutes. The pain was perceived to be 10 out of 10 and was described as an inflated balloon that was squeezing from the inside out. The paramedics found the patient in his house hypotensive and lying in a right lateral decubitus position. The patient was given a bolus of normal saline and his blood pressure responded appropriately. Upon examination, the patient claimed to of had a two-year history of worsening mild midepigastric and left upper quadrant abdominal pain and was seen by his primary care provider whom prescribed him a proton pump inhibitor (PPI) which partially relieved some symptoms. In addition, the patient complained of being nauseated, feeling bloated after meals, having a history of bloody stools one week prior to admission, and increased pain after eating. He denied vomiting or early satiety. Vital signs on admission were stable.

Past medical history included gastroesophageal reflux disease (GERD), hyperlipidemia and impaired fasting glucose. His medications consisted of a PPI, H2-blocker and simvastatin. The only past surgical history was an appendectomy. The patient was a truck driver for several years. He denied alcohol, drug use, and smoked approximately ½ packs of cigarettes a day for the past 40 years. He reported his two nieces having an unspecified type of cancer but no other known family history of cancer or heart diseases. Review of systems was negative except for gastrointestinal complaints as described above. The patient denied weight loss, fatigue, fever, shortness of breath, and/or chest pain. On physical examination, the abdomen was hard, guarding was noted, and there was direct and rebound tenderness noted in all four quadrants but worse in the epigastric area.

On admission, the patient had a complete laboratory workup involving all systems. The abnormalities found mildly decreased were hemoglobin of 12.9 g/dl, hematocrit of 39.6%, albumin of 3.1 g/dl, and sodium of 132 mEq/l. The only abnormalities found mildly increased was the red blood distribution width of 16%, white blood cell count of 15,100 ul, corrected calcium of 11.32 mg/dl, glucose of 129 mg/dl, prothrombin time of 12.2 seconds, and the international ratio of 1.2 seconds. Stool guaiac was negative.

**Figure 1.**
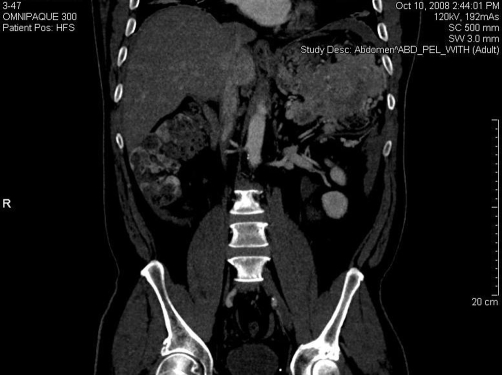
Computed tomography (CT) of the abdomen and pelvis. A large 4.7 centimeter (cm) lobulated hypodense mass is shown in the region of the porta hepatis likely secondary to the tumor mass.

Computed tomography (CT) of the abdomen and pelvis on admission revealed a large 4.7 centimeter (cm) lobulated hypodense mass in the region of the porta hepatis probably arising from the liver with the possibility of adjacent morphologic tubular lymph nodes or satellite lesions (Figure 1). Extending from the lobulated mass of the liver was a broad lobulated 10 cm band of density just deep to the rectus muscle extending from the upper abdomen to the level below the iliac crest, which possibly represented extraperitoneal infiltration. Additionally, an enhancing lesion was seen in the caudate lobe of the liver that was most likely metastasis. Blood was noted in both paracolic gutters, and there was a pancake-type density along the anterior abdominal wall, which was most likely blood that extended into the right pelvis representing omental caking. After review of the CT, serum measurements of Cancer antigen (CA) 19-9, Carcino embryonic antigen (CEA), and Alpha-fetoprotein (AFP) were found to be within normal limits. Serum Helicobacter pylori was positive. Colonoscopy and esophagastroduodenoscopy was recommended after review of the CT and findings were mild antral gastritis and a nearly obstructing mass in the descending colon. Recommendations were to rule out other malignancies and surgical intervention. An acute abdomen series with chest radiograph was consistent with the CT with additional findings of patchy and streaky infiltrate of the right and left lung bases and question of the mass in the left upper quadrant displacing the stomach medially. There was no evidence of acute obstruction or air under the diaphragm. Positron emission tomography scan was performed to evaluate for metastases and results showed increase uptake in the left upper quadrant, and about 4 cm of caudate lobe of the liver.

**Figure 2.**
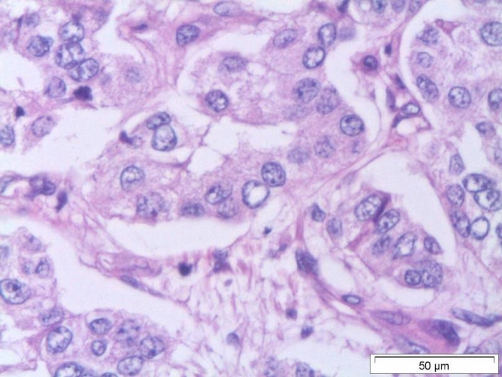
Histological demonstration of the well-differentiated neuroendocrine carcinoma. Pathology slides from the tumourus mass showing a well-differentiated neuroendocrine carcinoma. The architecture is consistent with the insular and trabecular morphologies, and the Ki-67 index is approximately 30% positive.

Utilizing CT guidance, biopsy of the large mass in the left upper quadrant was performed by Interventional Radiology on the same day of admission. Pathology results two days later showed a well-differentiated neuroendocrine carcinoma. The architecture revealed insular and trabecular morphologies, and the Ki-67 index was approximately 30% positive (Figure 2). Microscopic sections showed nested epithelial cells with moderately increased mitotic activity, nuclear atypia, irregular nuclear contours and hyperchromasia. Immunohistological stains were performed with the following results: the epithelial component was positive for cytokeratin AE1/AE3, neuroendocrine marker synaptophysin was positive, neuroendocrine marker chromogranin-A was positive, neuroendocrine marker neurone specific enolase was positive, and CA 19-9 was negative.

**Figure 3.**
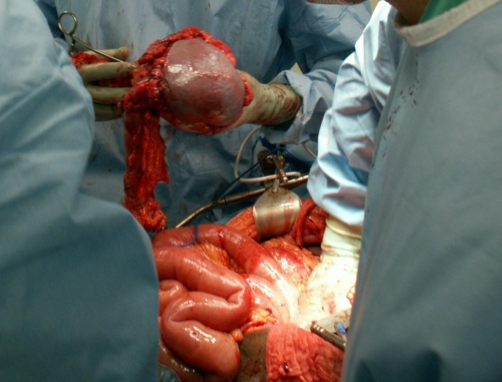
Exploratory laparotomy. Distal pancreatectomy, splenectomy, partial gastrectomy, left colectomy, and resection of the caudate lobe of the liver were performed during the surgical procedure.

On hospital day number six the surgery service performed a diagnostic laparoscopy, exploratory laparotomy with distal pancreatectomy, splenectomy, partial gastrectomy, left colectomy, and resection of the caudate lobe of the liver (Figure 3). Pre and postoperative diagnoses were well-differentiated neuroendocrine tumor of the pancreatic tail with metastasis to the caudate lobe of the liver. After diagnostic laparoscopy revealed a moderate amount of blood around the peritoneal cavity and metastasis to the liver it was necessary to convert to exploratory laparotomy. Blood was aspirated from the abdominal cavity and sent to cytology, which later revealed numerous neutrophils admixed with red blood cells yet no malignant cells. General inspection of the peritoneal cavity revealed a tumor in the caudate lobe as well as a large 10 cm mass that was fixed in the left upper quadrant. The tumor itself was quite vascular and was surrounded by varices, which was most likely the cause for the blood throughout the abdomen as well as within the lesser sac. Additionally, a periaortic lymph node appeared grossly positive and was then dissected and sent for analysis. There was no evidence of metastases in the pelvis, small bowel, or omentum. The diaphragm was not involved or infiltrated by the tumor and neither was the left kidney or adrenal glands.

Overall, the surgery was successful. At the end of the procedure two 10 ml Jackson-Pratt drains were inserted into the lesser sac and left upper quadrant.

**Figure 4.**
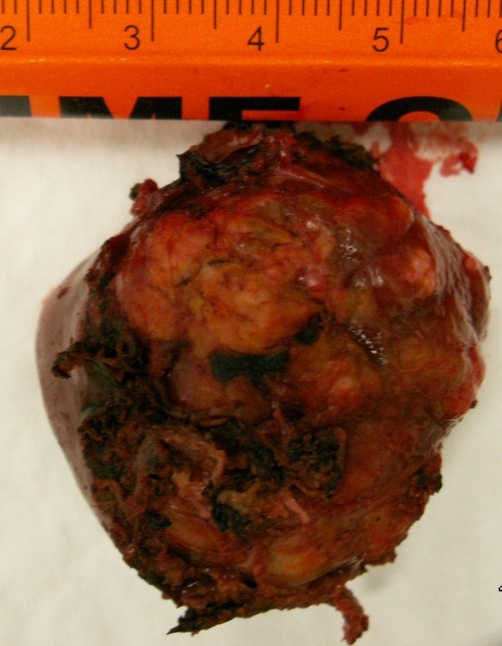
Gross specimen of the liver. One of the specimens removed from the body are shown here which presents the caudate lobe of the liver measured 4.0 by 3.5 by 2.5 cm.

**Figure 5.**
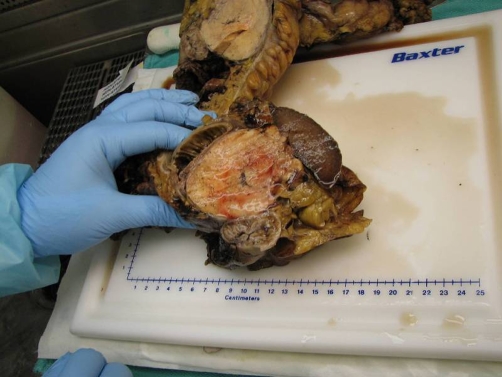
Splenic tissue. This histological picture is showing the tumor compressing the capsule of the spleen without any direct invasion.

Gross specimens were sent to pathology in three parts: frozen section of the periaortic lymph node measured 1.0 by 0.8 by 0.4 cm, the caudate lobe of the liver measured 4.0 by 3.5 by 2.5 cm, and the massive left upper quadrant tumor that consisted of an en bloc resection of the spleen, partial transverse colon, partial small bowel, pancreatic tail, partial distal stomach, adipose tissue, and lymph nodes. The spleen measured 12 by 7.5 by 2.5 cm, the colon measured 33 cm in length and 4 cm in diameter, the pancreatic tail measured 12 by 8 by 6 cm, a portion of the small bowel measured 15 cm in length and about 3 cm in diameter, and a small portion of the distal stomach measured 9 by 6.5 cm (Figure 4). The massive tumor invaded into the serosa of the transverse colon, distal stomach, and small bowel. The tumor appeared to compress the capsule of the spleen, however, no direct invasion was identified (Figure 5). After sectioning of all the involved organs it was noted that the tumor originated from the pancreas. Within the adjacent adipose tissue, multiple lymph nodes ranging in size from 0.3 to 1.0 cm were analyzed.

**Figures 6 & 7.**
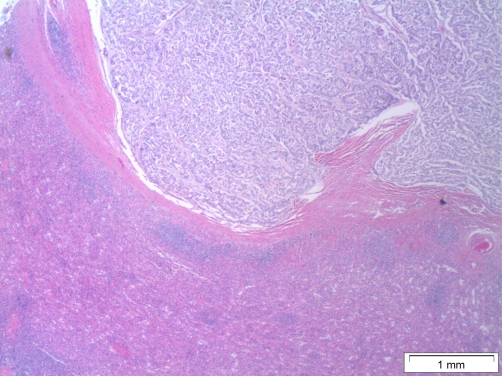

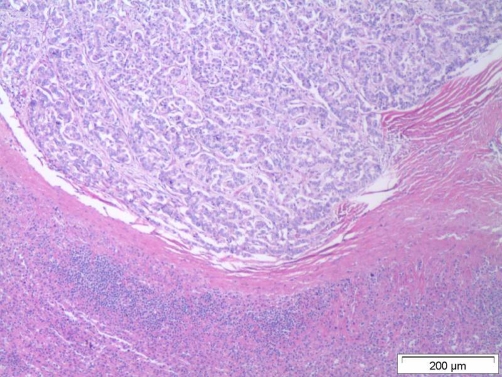
Microscopic sections of the caudate lobe of the liver. These pictures are demonstrating metastatic well-differentiated neuroendocrine carcinoma in different magnifications (exact numbers are mentioned in the figures).

Microscopic sections were analyzed and found the perioarotic lymph node to be negative for metastatic carcinoma. Microscopic sections of the caudate lobe of the liver showed metastatic well-differentiated neuroendocrine carcinoma (Figures 6 and 7). Sections of the massive left upper quadrant mass showed invasive well-differentiated neuroendocrine carcinoma characterized by cells with high nuclear to cytoplasmic ratio, irregular nuclear contours and hyperchromasia arranged in trabecular and insular patterns. There were 21 of 21 lymph nodes positive for metastatic carcinoma. Margins of resection were free of tumor. Tumor node metastasis (TNM) staging was T4, N1, M1.

Overall, the patient tolerated the procedure and hospital stay well. During the course of hospitalization his pain was well controlled with morphine, and his glucose was well controlled after being weaned off of an insulin drip and placed on a sliding scale. Vaccinations for encapsulated organisms including pneumococcal, *Streptococcus pneumoniae*, *Haemophilus influenza* and *Neisseria meningitides* were administered. Twenty days post-admission the patient was discharged from the hospital with instructions not to lift more than 15 pounds, and to follow-up with surgery in one week.

## Discussion

When the initial work-up, including physical examination, laboratory and radiographic studies fails to identify the primary site of a tumor with symptoms of referable to a metastatic site, light microscopic evaluation of biopsy material is necessary. Cancer of unknown primary site is a common entity and based on five histological categories: adenocarcinoma, poorly-differentiated carcinoma, poorly-differentiated neoplasm, squamous cell carcinoma, and neuroendocrine carcinoma [1]. Neuroendocrine carcinoma accounts for less than 5% of cancers of unknown primary site [2]. With that in mind, we present the clinical, pathological, histological and diagnostic features of well-differentiated neuroendocrine carcinoma which emphasis placed on pancreatic neuroendocrine tumors as seen in this case report.

Neuroendocrine carcinomas falls into three broad categories: poorly, moderately and well-differentiated neuroendocrine carcinomas. Poorly-differentiated neuroendocrine carcinomas usually have a high grade malignancy and are characteristically small cell undifferentiated or morphologically anaplastic by light microscopy [3]. Moderately-differentiated neuroendocrine carcinomas, such as atypical carcinoid tumors, manifest cellular pleomorphism, nuclear atypia, frequent mitosis, and necrosis [4]. In contrast, well-differentiated neuroendocrine carcinomas have variable and most often indolent biologic behaviors [5]. Histologically, well-differentiated neuroendocrine carcinomas show uniform and small cells organized in organoid or trabecular architecture [6]. Subtypes of well-differentiated neuroendocrine carcinomas include typical carcinoid tumors, pancreatic islet cell (neuroendocrine) tumors, paragangliomas, pheochromocytomas, and medullary thyroid carcinomas [5].

With well-differentiated neuroendocrine carcinomas encompassing such a broad spectrum of neoplasms it is pertinent to further classify them depending on whether they have a neural or an epithelial origin. Paragangliomas are of neural origin and the epithelial group can be subdivided into the remaining types of neuroendocrine carcinomas [7]. In our case, the epithelial component was illustrated microscopically by areas of nested and trabecular (neuroendocrine) growth, large cell size, irregular nuclear contours, and coarse nuclear chromatin. In addition, the immunohistochemical documentation showed immunoreactivity for markers of epithelial and neuroendocrine differentiation with positivity for cytokeratin AE1/AE3, synaptophysin, chromogranin, and neurone specific enolase [8]. Recognition of this unusual morphologic appearance is of importance to avoid mistaking these lesions for other types of malignant neoplasms.

The majority of well-differentiated neuroendocrine carcinomas involve the gastrointestinal tract, and therefore are referred to as gastroenteropancreatic neuroendocrine carcinomas, however neuroendocrine carcinomas have been found to occur in almost every organ in the body [9]. Gastroenteropancreatic neuroendocrine tumors are relatively rare, and pancreatic endocrine tumors are even more rare [10]. In our case the tumor originated in the pancreas and further infiltrated the transverse colon, small bowl, stomach, and lymph nodes within the adipose tissue. Literature review reported that low-grade neuroendocrine carcinomas, such as metastatic carcinoid or pancreatic endocrine tumors, are occasionally found at metastatic sites without an obvious primary site but almost always involve the liver [11]. Following the literature, in this case of pancreatic neuroendocrine origin there was metastasis specific to the caudate lobe of the liver.

Pancreatic endocrine tumors or islet cell tumors may be nonfunctional or functional depending on whether or not the tumor was secreting excessive amounts bioactive substances such as insulin, gastrin, somatostatin, vasoactive intestinal peptide, or glucagon and further causing associated symptoms [12]. Initially the patient presented with hypercalcemia, impaired fasting glucose, and GERD but due to urgency to undergo surgery and expected delay of the pathology it was not evident to look into these conditions until after the surgery. Therefore, we recommend looking into abnormal laboratory values or diseases before surgery to evaluate for associations related to neuroendocrine syndromes. In this case we cannot be certain if the patient was suffering from functional or nonfunctional well-differentiated neuroendocrine carcinoma of the pancreas. Since part of the stomach was removed it was difficult to determine if the GERD that the patient was suffering from preoperatively was due to high gastrin levels and/or if the tumor infiltration pushing upwards on the stomach was causing the acid reflux symptoms. In addition, it was difficult to determine postoperatively if the hypercalcemia, renal stone and/or the impaired glucose fasting levels as seen in our patient were attributed to a functional versus nonfunctional pancreatic endocrine tumor.

We looked into reasons of why metastasis occurred in the caudate lobe of the liver and not the other three lobes. Interesting, the caudate lobe is the only part of the liver that has hepatic venous drainage encompassing a few sizeable and several smaller branches that flow directly into the inferior vena cava [13]. Whether or not that is relevant it was interesting because even though many studies have reported tumors frequently infiltrating the parenchyma of the caudate lobe and/or its bile ducts it is still a rare entity [14,15].

Although there is abundant literature on neuroendocrine carcinomas [5,7,9], to our knowledge, there has not been a case presentation of neuroendocrine carcinoma evolving from the pancreas with metastasis to the caudate lobe of liver of such massive size. We have included many illustrations to show the severity of this case and to emphasize the importance of examining patients thoroughly especially when vague symptoms are chronic. When primary pancreatic cancer is diagnosed, 95% of cases are adenocarcinoma in origin and the remaining 5% are due to neuroendocrine carcinoma [16]. In contrast to neuroendocrine carcinomas, adenopancreatic cancers are generally very aggressive [17]. A study assessed the clinical relevance of the World Health Organization and TNM classifications in patients with pancreatic neuroendocrine carcinomas and found that the five-year survival rate to be 44% [18]. Another study found the five-year survival rate to be 58.3% and concluded that patients with well to moderately differentiated tumors had a better (63.9%) five-year prognosis than those with poorly differentiated tumors (28%) [19]. Furthermore, another study found the five-year survival rate to be 80% for aggressive surgical resection for pancreatic neuroendocrine tumors [20]. Regardless of the survival rate, the prognosis seems to be determined by various biological factors. With regard to the principles of surgical oncology, tumor-free resection margins are fundamental and radical surgical procedures are justified in selected patients.

## Conclusions

Well-differentiated neuroendocrine carcinomas have variable and most often indolent biologic behaviors, and microscopically show uniform and small cells organized in organoid or trabecular architecture. Biopsy and immunohistology is necessary to distinguish the various types of neuroendocrine carcinomas whether they are epithelial versus neural origin or poor versus well-differentiated and additional laboratory workup should be done to differentiate between functional and nonfunctional types of pancreatic endocrine tumors which may be associated with neuroendocrine syndromes. More importantly, neuroendocrine carcinomas of all kind have been capable of local recurrence and distant metastasis, and therefore, close clinical correlation and appropriate treatment are important factors attributed to improving the overall survival rate.

## Abbreviations

AFP: alpha-fetoprotein
CA: cancer antigen 19-9
CEA: carcino embryonic antigen
CT: computed tomography
CUP: cancer of unknown primary
GERD: gastroesophageal reflux disease
TNM: tumor node metastasis
